# Comparison of Efficiency of Closed Kinetic Chain Exercises Versus Proprioceptive Exercises in Improving Balance and Gait in People With Hemophilia: Protocol for a Randomized Controlled Trial

**DOI:** 10.2196/66770

**Published:** 2025-04-24

**Authors:** Tugce Poyraz Isleyen, Ela Tarakci, Gokce Leblebici, Ipek Yeldan, Bulent Zulfikar

**Affiliations:** 1 Department of Physiotherapy and Rehabilitation Institute of Graduate Studies Istanbul University-Cerrahpaşa Istanbul Turkey; 2 Department of Physiotherapy and Rehabilitation Faculty of Health Sciences Bahçeşehir University Istanbul Turkey; 3 Department of Physiotherapy and Rehabilitation Faculty of Health Sciences Istanbul University-Cerrahpaşa Istanbul Turkey; 4 Department of Physiotherapy and Rehabilitation Faculty of Health Sciences Istanbul Medeniyet University Istanbul Turkey; 5 Hereditary Bleeding Disorders Unit Institute of Oncology Istanbul University Istanbul Turkey

**Keywords:** hemophilia A, hemophilia B, exercise, gait, postural balance, efficiency, hemophilia, proprioceptive exercises, balance, protocol, randomized controlled trial (RCT), bleeding disorder, protein deficiencies, blood clotting, quality of life, young adult, teenager

## Abstract

**Background:**

Inherited bleeding disorders involve prolonged bleeding due to clotting protein deficiencies, with hemophilia A and B being the most common types. The severity of bleeding in people with hemophilia depends on the deficient factor level. Treatment includes coagulation factor concentrates, nonreplacement preparations, gene therapies, and physiotherapy, whereby bleeding is prevented, symptoms are reduced, and the quality of life is improved. Closed kinetic chain exercises improve joint stability and neuromuscular control by stabilizing the proximal base, making them favored in musculoskeletal rehabilitation. Proprioceptive exercise training improves the sensorimotor system’s adaptability and injury prevention through tailored programs involving progressively complex movements and surfaces.

**Objective:**

The aim of this study is to investigate the effects of closed kinetic chain exercises and proprioceptive exercise training on improving balance and walking in people with hemophilia.

**Methods:**

This study is a 3-arm, parallel-group randomized controlled trial with 63 people with hemophilia aged 13-25 years who meet the inclusion criteria. The primary outcome measures are medio-lateral swing, anterior-posterior swing, walking speed, and Hemophilia Joint Health Score. Secondary outcome measures are kinematic assessment of gait, one-leg stand test, 6-minute walk distance (6MWD) test, proprioception assessment, and the Functional Independence Score in Hemophilia. Participants will be evaluated with the Biodex balance system for postural sway, 10-meters walking test for gait speed, Hemophilia Joint Health Score for joint health, Kinovea 2D motion analysis for kinematic evaluation of gait, one-leg stand test for balance measurement, 6MWD for functional capacity, digital goniometer for proprioception, and Functional Independence Score in Hemophilia for functional independence. Participants will be randomly assigned to a closed kinetic chain exercise group, a proprioceptive exercise group, or a control group. All participants in exercise training groups will receive a 30-minute education session on joint protection techniques and energy conservation prior to the first exercise session. Closed kinetic chain exercises will include progressive lower limb exercises of approximately 45 minutes each session. Proprioceptive exercise training will focus on vibration training; reposition exercises and proprioception exercises will be administered to increase proprioceptive input for the same duration as the other group. The control group will receive no intervention. All participants will undergo 24 exercise sessions (2 days a week for 12 weeks). After the treatment, the initial measurements will be repeated.

**Results:**

This study began in September 2023 and is scheduled to be completed in May 2025. A total of 34 participants have completed the study to date.

**Conclusions:**

This study will investigate the effects of 2 different exercises on functional parameters in people with hemophilia. The effects of different exercise protocols on parameters such as postural sway, walking speed, and joint health will be evaluated. It is predicted that both exercise methods may have positive effects on balance and gait.

**Trial Registration:**

Clinical Trials.gov NCT05879549; https://clinicaltrials.gov/study/NCT05879549

**International Registered Report Identifier (IRRID):**

DERR1-10.2196/66770

## Introduction

Inherited bleeding disorders are rare diseases characterized by prolonged bleeding time due to deficiencies in protein cofactors and enzymes involved in blood clotting [1]. Among coagulopathies, hemophilia A, caused by a deficiency of factor VIII, and hemophilia B, resulting from a deficiency of factor IX, are the most common [2]. The severity of hemophilia is determined by the levels of the clotting factors in the blood, and is classified as mild, moderate, or severe hemophilia [3]. In mild hemophilia, bleeding occurs due to trauma, surgical procedures, and dental intervention. In moderate hemophilia, recurrent musculoskeletal bleeding is observed. In severe cases, spontaneous bleeding is also common [1].

In people with hemophilia, the location, timing, and severity of the clinical symptoms and findings vary depending on the level of the deficient factor. Bleeding in the musculoskeletal system is the most common type of bleeding, with 70%-80% occurring in the joints and about 15% occurring in the muscles [4]. The most frequently affected joints are the knee, ankle, and elbow [2]. Recurrent hemarthroses in people with hemophilia cause hypertrophy of the synovial membrane, making the joint more susceptible to further bleeding and increasing the risk of hemophilic arthropathy [5].

Current pharmacological treatments for hemophilia include standard or extended half-life factor preparations, therapies balancing the nonfactor coagulation system, intravenous or subcutaneous applications, and gene therapies that have been licensed in recent years [5,6]. In addition to pharmacological treatment, various physiotherapy methods are applied to prevent new bleeds and alleviate existing symptoms. The goals of physiotherapy and rehabilitation are to relieve pain, increase joint range of motion, prevent muscle atrophy, improve functional ability, reduce the frequency of joint bleeds, and enhance the quality of life [7].

According to the closed kinetic chain exercise model, consecutive rigid segments are connected by intermediary segments [8]. The terminal segment is fixed in this exercise model, and body weight resistance is provided. The advantage of closed kinetic chain activities is that they create stability in the proximal segment, providing a more solid base for distal mobility or ambulation, resulting in increased joint stability and neuromuscular control [9]. Closed kinetic chain exercises have been preferred in the rehabilitation of many musculoskeletal problems over the past 20 years [8,10].

Proprioception is a neural process that includes the body’s ability to respond to sensory inputs from the environment [11]. Proprioceptive exercise training can enhance the sensorimotor system’s ability to adapt to changing environments, thereby protecting the body from injuries [12]. Proprioceptive exercise training includes various exercises tailored to the patient’s condition, focusing on transitioning from hard to softer surfaces, stable to unstable surfaces, simple to more complex movements, supported to unsupported movements, and different joint angles [13]. Sensorimotor training programs for lower extremity proprioception exercises include active joint repositioning exercises, coordination exercises, muscle performance, balance or unstable surface training, plyometric training, and vibration training [14].

Due to the frequent occurrence of hemarthroses in the lower extremity joints, changes in balance and walking parameters can be observed in people with hemophilia. A previous study has shown that the restricted rolling behavior of the foot in individuals with hemophilia, which may be caused by movement limitations and pain, can alter the balance between different foot areas [15]. Another study reported that changes in gait patterns in boys with hemophilia are associated with altered physical function performance [16].

Research [17,18] has demonstrated that both proprioceptive and closed kinetic chain exercises have beneficial effects on functional abilities in patients with lower extremity impairments. However, comparative studies evaluating the efficacy of these 2 exercise modalities specifically in people with hemophilia are yet to be conducted. This study aims to investigate the effects of closed kinetic chain exercises and proprioceptive exercise training approaches on improving balance and walking in people with hemophilia.

## Methods

### Participant Selection

The sample size was calculated as 18 individuals for each group using the G*Power program (version 3.1.9.4; Heinrich Heine University) with 90% power and .05 type 1 error using the medio-lateral swing effect size value as reference in the balance assessment in the study by Tat et al [19]. Considering that there might be individuals who would not complete the study, a total of 63 participants, that is, 21 individuals for each group, will be included.

The inclusion criteria of the participants in this study were as follows: (1) diagnosed with hemophilia A and hemophilia B, (2) have a blood factor level below 5%, (3) is 13-25 years of age, (4) have a history of bleeding in the lower extremities, (5) have a target joint, (6) have a total lower extremity Hemophilia Joint Health Score ≥3, and (7) is receiving prophylaxis (factor support to prevent bleeding). The exclusion criteria were as follows: (1) individuals having undergone lower extremity surgery in the last 6 months, (2) having BMI>30 kg/m^2^, (3) having an inhibitor, (4) using a walking aid, and (5) being involved in any physiotherapy or exercise program in the last 6 months.

### Ethics Approval

This study was approved by Istanbul University-Cerrahpaşa ethics committee (E-74555795-050.01.04-689576; May 3, 2023). The Declaration of Helsinki will guide this study. After eligibility is confirmed, written and verbal information about the study will be provided to all the participants. Then, the participants will sign a written informed consent form stating that they agree to participate in the research. The informed consent form also states that participation in the study is free and voluntary. No additional fees will be charged to the participants, and no payment will be made. The personal data and identity information of the participants will be kept confidential. Participants have the right to withdraw from the study at any time. The data will be used purely to contribute to science without any profit motive (Multimedia Appendix 1). At the same time, the data can be statistically analyzed without secondary approval when necessary. The trial intervention is similar to other clinical practices; therefore, we consider that risks from the trial are minimal. Additionally, exercise training approaches in the intervention group will be applied with the same protocol and duration to the participants in the control group at the end of the study.

### Study Design

**Figure 1:**
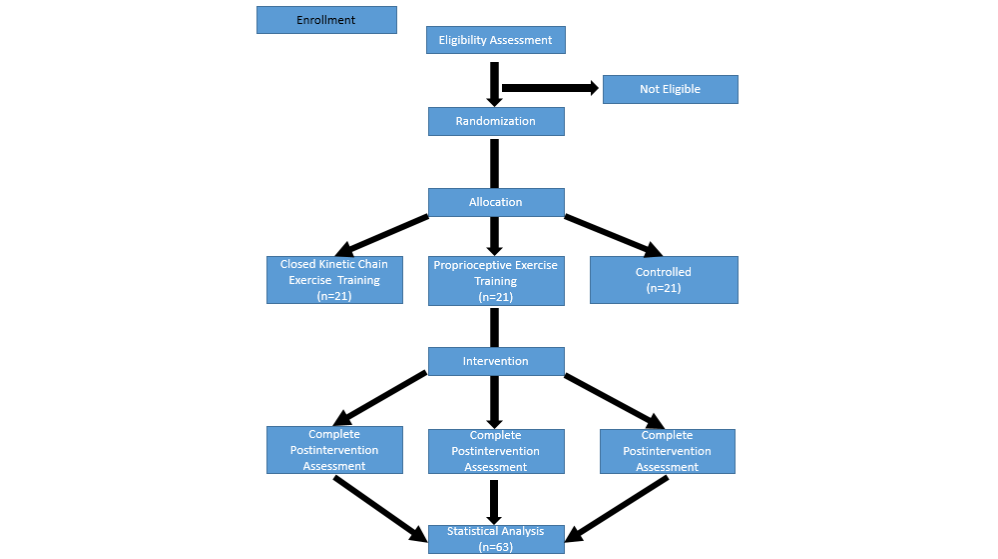
Flowchart in this study.

This study has been registered with Clinical Trials.gov (NCT05879549). It is a 3-arm, parallel-group, single-blinded randomized controlled trial. This trial has been reported according to CONSORT (Consolidated Standards of Reporting Trials) guidelines (Multimedia Appendix 2) and the SPIRIT (Standard Protocol Items: Recommendations for Interventional Trials) checklist (Multimedia Appendix 3). This study will be performed in Istanbul University-Cerrahpaşa Laboratory. A permit has been obtained from the institution. The demographic characteristics of the participants (age, height, weight, diseases, etc) will be recorded. Participants will be divided into 3 groups using a simple randomization method according to the order of arrival (1:1:1) with a web-based randomizer. Participants do not know the exercise group that they will be included in nor will they know the differences between the exercises. The flowchart of this study is shown in Figure 1.

### Outcome Measures

The primary outcome measures are medio-lateral swing, anterior-posterior swing, walking speed, and Hemophilia Joint Health Score. Secondary outcome measures are kinematic assessment of gait, one-leg stand test, 6-minute walk distance (6MWD), proprioception assessment, and Functional Independence Score in Hemophilia. All patients will have completed their factor replacement 1 hour before the assessment section.

#### Postural Sway

**Figure 2:**
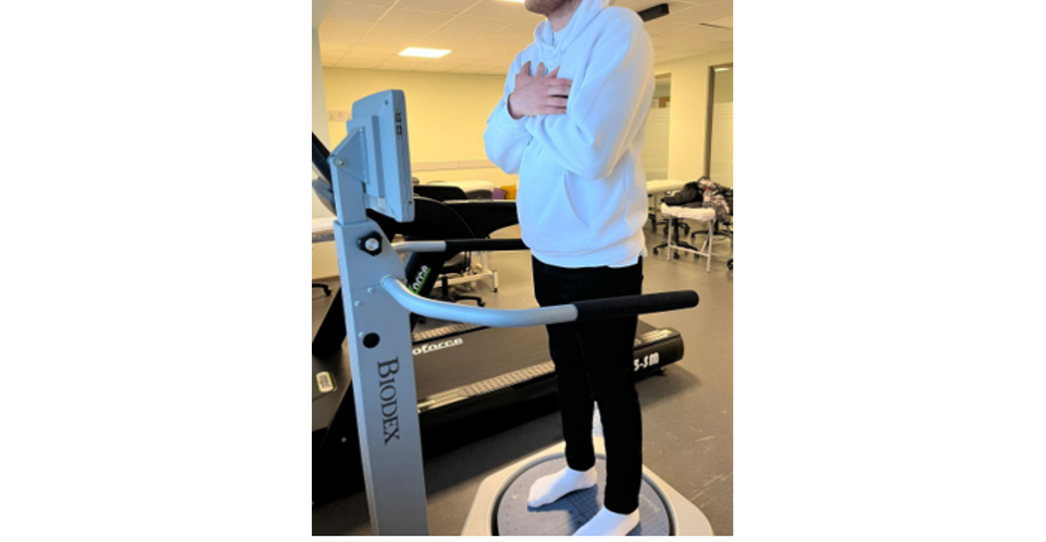
Biodex balance system.

Postural sway will be evaluated using the Biodex balance system, which is a reliable method (Figure 2) [20]. Postural sway, center of pressure, center of gravity, anterior-posterior, and lateral sway will be evaluated with eyes closed and open.

**Figure 3:**
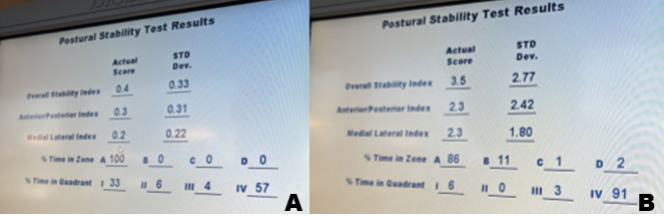
Example of postural sway. (A) Postural sway with eyes open. (B) Postural sway with eyes closed.

Patients will be informed about the purpose and performance of the tests before the assessment. Each test will be repeated 3 times. A lower sway index in all tests indicates a better postural stability, while an increase in the sway index indicates a decrease in postural stability (Figure 3).

#### Joint Health

The Hemophilia Joint Health Score is a hemophilia-specific score, including measurements of muscle strength; edema; crepitation; range of motion of bilateral elbow, knee, and ankle joints; and assessments related to walking [21]. The maximum score to be obtained from each joint is 20, and the maximum score to be obtained from the global gait score is 4. The maximum score to be obtained from the assessment tool is calculated as 124, and a high score indicates poor joint health. The parameters to be evaluated within the scope of the score are as follows: swelling, swelling duration, muscle atrophy, flexion and extension loss, joint pain, muscle strength, and global gait score.

#### Kinematic Evaluation of Gait

**Figure 4:**
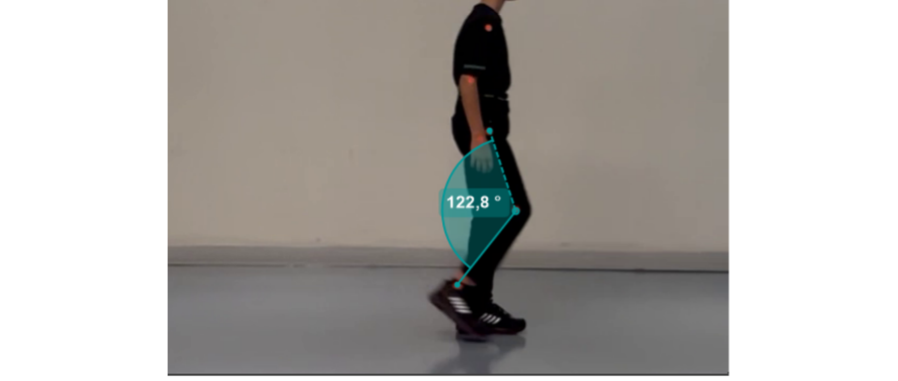
Screenshot of Kinovea 2D motion analysis software.

The spatiotemporal parameters of hip, knee, and ankle joints and walking speed will be analyzed during walking using Kinovea 2D motion analysis software (Figure 4). In order to evaluate the lower extremity joint angles during walking, video footages will be taken with 2 cameras fixed with a tripod placed laterally and anteriorly [22]. The fifth metatarsophalangeal joint, lateral malleolus, lateral femoral condyle, and trochanter major will be marked using the reflector tape (adhesive circular tapes that contrast with the background).

#### Balance

The one-leg standing balance test will be evaluated with eyes open and closed [23]. The participant lifts one foot with eyes open so that it does not touch the support leg and tries to maintain this position. The individual is expected to maintain this position for a maximum test duration of 30 seconds. The time he remains on one leg will be recorded.

#### Functional Capacity

The 6MWD test will be applied for the evaluation of functional capacity. The patient is asked to walk as far as possible at their own walking pace for 6 minutes in a 30-meter straight corridor. Before starting the test, the patient rests in a sitting position for 10 minutes in the corridor where the test will be performed. At the end of the test, 6MWD is recorded in meters [24].

#### Proprioception

A digital goniometer will be used to assess proprioception. Proprioception assessment will be performed for knee and ankle joints. During the knee joint assessment, the patient will be positioned from the bed with feet hanging down and knees in 90° flexion. The pivot of the digital goniometer will be placed on the lateral condyle of the femur, and the knee will be brought to the previously determined 15° and 30° extension angles. The patient will be asked to perceive the position of this angle by waiting for 10 seconds with eyes open. Then, the patient will be asked to actively repeat these angles 3 times for both knees with eyes closed. For ankle proprioception measurement, the patient will be placed in a long sitting position on the bed. A neutral position will be provided for the ankle; the lateral malleolus of the fibula will be taken as the pivot point and the goniometer will be placed on the pivot point. The fixed arm of the goniometer will be kept parallel to the lateral midline of the fibula, and the movable arm will be placed to follow the lateral midline of the 5th metatarsal bone. The goniometer will be brought to an angle of 20°, and the patient will be asked to make a plantar flexion movement of the ankle and hold it at this angle for 5 seconds with the eyes open. Then, the patient will be asked to return to the starting point and find the target angle, that is, the range of motion of 20°, with the eyes closed. The measurement will be repeated 3 times for both ankles with the same method [25].

#### Functional Independence

The Functional Independence Score in Hemophilia has been developed as an assessment tool used to measure the functional ability of the patient with hemophilia. The patient is asked to score their level of independence in 8 activities under 3 main headings related to activities of daily living. These activities are eating or self-care, bathing, dressing, transfers, squatting, walking, climbing stairs, and running. The patient will be asked questions and asked to score between 1 and 4. The total score will be recorded [26].

### Intervention

All participants will be divided into 3 groups. Group 1 is the closed kinetic exercise group, group 2 is the proprioceptive exercise group, and group 3 is the control group. Participants in group 1 and group 2 will receive exercise training twice a week for 12 weeks for a total of 24 sessions of 45 minutes each. The control group will not receive any intervention. Exercise training will continue under the supervision of the physiotherapist. Patients will have completed their factor replacement on the day of exercise. If any signs of bleeding are observed, the exercise session will be terminated. Following randomization, all participants in exercise training groups will receive a 30-minute education session on joint protection techniques and energy conservation prior to the first exercise session. This training will cover the goals and basic principles of joint and energy education; correct and incorrect posture characteristics; proper standing, sitting, and lifting techniques; the benefits of correct posture; posture recommendations for different activities; hand protection methods; factors contributing to fatigue; and basic principles of energy management.

### Closed Kinetic Chain Exercise Training

**Figure 5:**
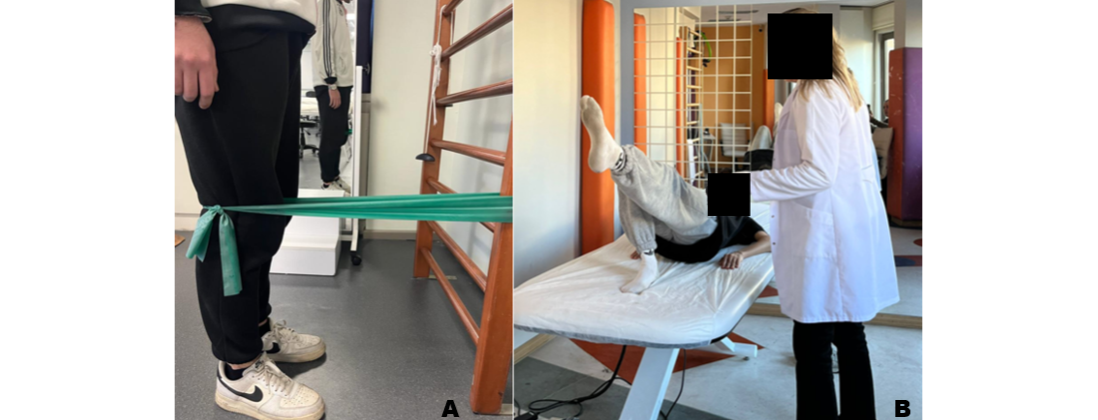
Examples of closed kinetic chain exercises. (A) Terminal knee exercise with resistance band. (B) Single leg bridge exercise.

Group 1 exercises will start with a 5-minute warm-up. The warm-up exercises include marching in place, shoulder circles, elbow stretching, seated knee flexion and extension, as well as stretching exercises targeting the hamstring and gastrocnemius muscles. Thereafter, 6-8 different closed kinetic chain exercises will be planned as 2-3 sets and 8-10 repetitions (Figure 5). Subsequently, the session will be finished by cooling down for 5 minutes and applying ice to the lower extremity joints for 15 minutes. Each session is expected to last approximately 40-45 minutes. Exercises will be progressively made more difficult once every 2 weeks. Exercise training will continue under the supervision of a physiotherapist 2 days a week for 12 weeks. The details of the closed kinetic chain exercise training and progression are given in Table 1.

**Table 1:** Closed kinetic chain exercises protocol.

			Repetitions (n)		Sets (n)
**Weeks 1-3**					
	Terminal knee extension (both sides)	10		2	
	Bridge	10		2	
	Bridge with hip adduction	10		2	
	Lunge (both sides)	10		2	
	Side lunge (both sides)	10		2	
	Mini squat	10		2	
	Step up (both sides)	10		2	
**Weeks 4-6**					
	Terminal knee extension (both sides)	10		3	
	Bridge	10		3	
	Bridge with hip adduction	10		3	
	Lunge (both sides)	10		3	
	Side lunge (both sides)	10		3	
	Mini squat	10		3	
	Step up (both sides)	10		3	
**Weeks 7-9**					
	Terminal knee extension with mild resistance band (both sides)	10		2	
	Bridge with mild resistance band	10		2	
	Single leg bridge	10		2	
	Lunge (both sides)	10		2	
	Side lunge (both sides)	10		2	
	Mini squat on unstable surface	10		2	
	Mini squat with hip adduction (both sides)	10		2	
**Weeks 10-12**					
	Terminal knee extension with mild resistance band (both sides)	10		3	
	Bridge with mild resistance band	10		3	
	Single leg bridge	10		3	
	Lunge (both sides)	10		3	
	Side lunge (both sides)	10		3	
	Mini squat on unstable surface	10		3	
	Mini squat with hip adduction(both sides)	10		3	

### Proprioceptive Exercise Training

**Figure 6:**
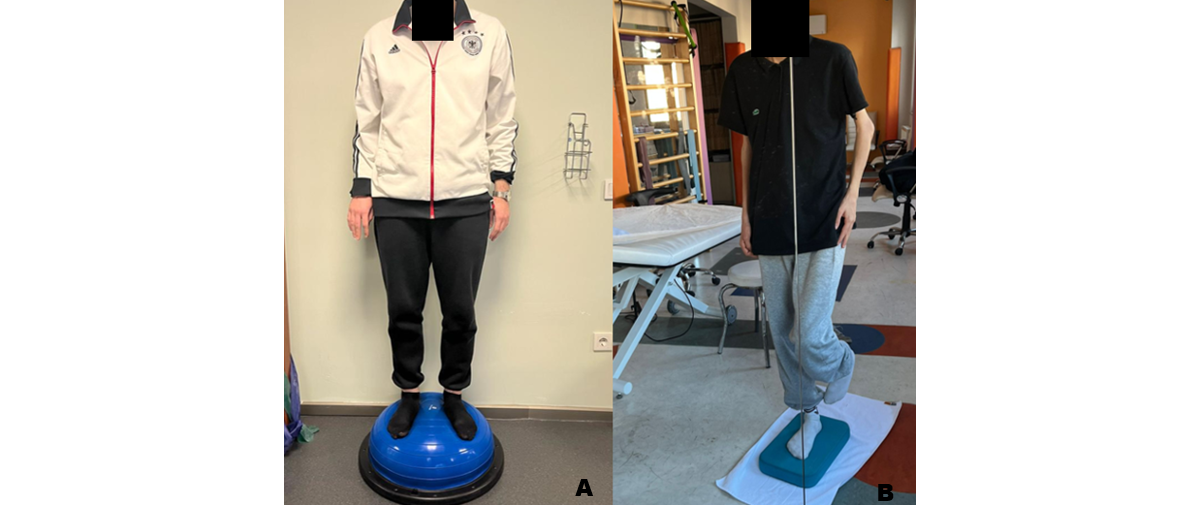
Examples of proprioceptive exercises. (A) Standing on a Bosu ball. (B) One-leg stand on a foam surface.

Proprioceptive exercise training will focus on sensorimotor training. In this training model, vibration training, reposition exercises, and proprioception exercises will be given to increase proprioceptive input (Figure 6).

Exercise training will start with the same 5-minute warm-up as in the closed kinetic chain exercise group and will continue with vibration application to the knee and ankle joints. Since damage or loss of mechanoreceptors due to degenerative events in the joint in people with hemophilia may adversely affect the balance and proprioception required in activities, there may be a decrease in lower extremity vibration sensation [27].

Vibration training will be applied to 4 points, that is, patellar tendon, medial knee joint, lateral knee joint (medial collateral ligament and lateral collateral ligament), and the upper part of the patella bone. The application to each point will start with 30 seconds, and the application time will be increased by adding 15 seconds every 2 weeks.

Then reposition exercises will be started. Kinetic reposition exercises will be performed on 3-way lines drawn on a white cardboard paper. One of the lines drawn on the white cardboard is straight, and the other 2 are lines drawn at 30° angles to the straight line, considering that the internal and external (axial) rotation of the tibia on the femur is 45° when the knee is at 90°. Numbers in centimeters will be written on the lines. The patient will be seated on the chair with 90° knee flexion and will be asked to put his or her foot on these lines and hold it for 10 seconds to take his or her foot to the point 10 cm away and learn that point. Afterwards, the patient will return to the starting point and will be asked to find the point he or she learnt with his or her eyes closed.

The program will be planned as 6-8 different proprioceptive exercises performed in 2-3 sets and 8-10 repetitions. Finally, the session will be finished by cooling down for 5 minutes and applying ice to the lower extremity joints for 15 minutes. Each session is expected to last approximately 40-45 minutes. Exercises will be progressively made more difficult once every 2 weeks. The details of proprioceptive exercise training and progression are given in Table 2.

**Table 2:** Proprioceptive exercises protocol.

		Duration or repetition (min or s)	Sets (n)
**Weeks 1-3**			
	Warm-up	5 min	N/A^a^
	Application of vibration (patellar tendon, medial, and lateral sides of the knee joint; both sides)	30 s	N/A
	Kinetic repositioning exercises (both sides)	All directions	5
	Standing on a balance board	10 s	2
	Standing on a Bosu ball	10 s	2
	One-leg stand	10 s	2
	Semitandem walking	10 s	3
	Cool down	5 min	N/A
**Weeks 4-6**			
	Warm-up	5 min	N/A
	Application of vibration (patellar tendon, medial, and lateral sides of the knee joint; both sides)	45 s	N/A
	Kinetic repositioning exercises	All directions	5
	Standing on a balance board	10 s	3
	Standing on a Bosu ball	10 s	3
	One-leg stand	10 s	3
	Semitandem walking	30 s	2
	Cool down	5 min	N/A
**Weeks 7-9**			
	Warm-up	5 min	N/A
	Application of vibration (patellar tendon, medial, and lateral sides of the knee joint; both sides)	60 s	
	Kinetic repositioning exercises (both sides)	All directions	10
	Eyes open and close standing on a balance board	15 s	2
	Eyes open and close standing on a Bosu ball	15 s	2
	Eyes closed one-leg stand	15 s	2
	One-leg stand on foam surface	15 s	2
	Tandem standing	15 s	2
**Weeks 10-12**			
	Warm-up	5 min	N/A
	Application of vibration (patellar tendon, medial, and lateral sides of the knee joint; both sides)	60 s	N/A
	Kinetic repositioning exercises (both sides)	All directions	10
	Eyes open and close standing on a balance board	20 s	3
	Eyes open and close standing on a Bosu ball	20 s	3
	Eyes closed one-leg stand	20 s	3
	One-leg stand on foam surface	20 s	3
	Tandem standing	20 s	3

^a^N/A: not applicable.

### Statistical Analysis

Data will be statically analyzed using SPSS (version 25.0; IBM Corp). Normality tests (visual and analytical) will be applied. Descriptive statistics will be presented as mean (SD) or frequency (percentage). The Shapiro-Wilk test will be used to assess the normality of data distribution. For normally distributed data, a one-way analysis of variance test will be applied for between-group comparisons, and a 2-sided paired sample *t* test will be used for within-group changes. For nonnormally distributed data, the Kruskal-Wallis test will be used for between-group comparisons, and the Wilcoxon test will be applied for within-group changes. In cases where between-group comparisons of normally distributed data show significant results, Tukey or Bonferroni corrections will be used as post hoc analyses to identify the source of the differences. For nonnormalized distributed data, Mann-Whitney *U* tests will be conducted for between-group comparisons. A *P* value <.05 will be considered statistically significant for all analyses.

## Results

This research received funding from the Turkish Scientific and Technological Research Institution as of September 1, 2023. From this date, cases began to be incorporated into the study. By April 2025, a total of 34 participants had completed the treatment phase. Once all enrolled participants have finished their treatment, data analysis will commence. The anticipated completion date for this study is May 2025.

## Discussion

This study will investigate the effects of 2 different exercise approaches on functional parameters in people with hemophilia. In this study, the effects of exercise protocols on parameters such as postural sway, walking speed, and joint health will be evaluated. The predicted main findings of this study suggest that both exercise approaches may positively affect functional parameters such as postural sway, walking speed, and joint health in individuals with hemophilia, with anticipated improvements in balance and gait.

Recurrent bleeding in the lower extremities of people with hemophilia may cause changes in gait. Fouasson-Chailloux et al [28] observed that patients with moderate hemophilia may have impairments in gait parameters if their joint health scores are poor [28]. Joint movement limitations in people with hemophilia may occur due to movement restrictions and pain sensation, and this may lead to decreased pressure in the forefoot center and shorter step length [28]. Fukuchi et al [29] reported that people with hemophilia walked with less range of motion in the hips and ankles compared to their healthy peers. In addition, a decrease in gait speed and step length and an increase in double support and swing phase duration were observed in patients with moderate or severe hemophilia [30]. Changes in the walking patterns of boys with hemophilia are associated with changes in physical function performance [15]. Deniz et al [31] reported that an 8-week structured exercise program can improve the kinematic parameters of walking. For these reasons, the effect of exercise programs on walking will be investigated in our study, and for this purpose, changes in walking speed and kinematic parameters will be evaluated using the Kinovea software (Joan Charmant, open source).

Recurrent bleeding is the major factor leading to hemophiliac arthropathy. Lower extremity arthropathies may cause balance problems. A previous study [19] where static posture was evaluated using Biodex similar to our study showed that medio-lateral oscillations increased in people with hemophilia. These changes were found to be associated with the clinical scores of the lower extremity joints. That study emphasized the importance of balance assessment and suggested that exercises to enhance medio-lateral stability should be incorporated into rehabilitation programs. Although lower extremity arthropathies may affect balance scores, worse balance scores have been reported in patients with and without hemophilic arthropathy compared to healthy individuals. For this reason, we incorporated methods from the literature known to positively impact balance parameters into our study.

Exercise training is one of the most commonly recommended methods to prevent and treat musculoskeletal problems that may develop in patients with hereditary bleeding disorders [15]. Traditionally, the most commonly used exercise types are aerobic exercises, strengthening exercises, aquatic exercises, and combined training. In our study, the effectiveness of customized exercise programs will be compared differently from that in existing literature. Chronic synovitis, cartilage damage, and subchondral bone damage due to recurrent joint bleeding in people with hemophilia may decrease proprioceptive information [32]. For this reason, one of the exercise groups that we planned was based on proprioception. The effect of progressively developed sensorimotor exercises on balance and gait parameters will be revealed.

Gönen et al [33] performed different exercise trainings for 21 patients for 12 weeks in people with hemophilia: 7 patients were included in the closed kinetic chain exercise training group, 7 in the conventional exercise group, and 7 in the control group. They concluded that closed kinetic chain exercise training was superior in proprioception and physical activity compared to conventional exercises for people with hemophilia A [33]. In our study, we aim to investigate the effects of closed kinetic chain exercises on balance and gait parameters. Additionally, by the end of the study, we will compare the outcomes of proprioceptive exercise training and those of closed kinetic chain exercises. This comparison will contribute to developing optimal exercise training programs for people with hemophilia.

Bleeding episodes induced by exercise in people with hemophilia have been reported as rare adverse events [15]. In our study, to prevent any bleeding during exercises, the exercises were scheduled on the days when patients received their factor replacement therapy.

A limitation of this study is the lack of long-term follow-up, which is particularly relevant, given that hemophilia is a chronic condition that predisposes individuals to ongoing musculoskeletal system challenges. The absence of extended outcome assessments limits the ability to evaluate the sustained effects of the exercise interventions on balance, gait, and joint health over time.

Our study will contribute to the literature as the first study comparing the protocols of closed kinetic chain exercises and proprioceptive exercises in people with hemophilia. We expect our study to be a pioneer in the future, especially in protocols that contribute to the improvement of daily life activities such as balance and walking, which are affected by bleeding.

## Appendix

Multimedia Appendix 1 Peer-reviewer report from the Turkish Scientific and Technological Research Institution.

Multimedia Appendix 2 CONSORT-EHEALTH (Consolidated Standards of Reporting Trials of Electronic and Mobile Health Applications and Online Telehealth) checklist (version 1.6.1).

Multimedia Appendix 3 SPIRIT (Standard Protocol Items: Recommendations for Interventional Trials) checklist.

## Abbreviations

6MWD: 6-minute walk distance
CONSORT: Consolidated Standards of Reporting Trials
SPIRIT: Standard Protocol Items: Recommendations for Interventional Trials

## Data Availability

The data sets generated during and analyzed during this study are available from the corresponding author on reasonable request.
