# Supplementary material for: Comparison of Efficiency of Closed Kinetic Chain Exercises Versus Proprioceptive Exercises in Improving Balance and Gait in People With Hemophilia: Protocol for a Randomized Controlled Trial
Source: JMIR Res Protoc. 2025 Apr 24;14:e66770. doi: 10.2196/66770 (PMC12062759; doi:10.2196/66770)
Supplement: Multimedia Appendix 1 [file resprot_v14i1e66770_app1.pdf]

T.C.
TÜRKİYE BİLİMSEL VE TEKNOLOJİK ARAŞTIRMA KURUMU BAŞKANLIĞI
Araştırma Destek Programları Başkanlığı

Sayı : B.14.2.TBT.0.06.03.02-161-354698 01/09/2023

Konu : 223S216 Numaralı Proje Karar Yazısı

Sayın Tuğçe POYRAZ İŞLEYEN

"1002-A Hızlı Destek Modülü" kapsamında Kurumumuza sunulan 223S216 numaralı ve "Hemofilik Bireylerde Denge Ve Yürümenin Geliştirilmesinde Kapalı Kinetik Zincir Egzersizlerine Karşın Proprioseptif Egzersizlerin Etkinliğinin Karşılaştırılması" başlıklı projenize ilişkin bilimsel değerlendirme süreci tamamlanmıştır.

Konunun uzmanı danışmanlar tarafından yapılan değerlendirmeler sonucunda proje önerinize destek verilmesine karar verildiğini bildirmekten memnuniyet duyarım (*).

Desteklenmesine karar verilen proje önerinizin ilgili mevzuat çerçevesinde, mali ve benzeri konularda değerlendirme çalışmalarına başlanmıştır. Süreç tamamlandığında projelere ait sözleşme ve diğer belgeler imzalanmak üzere tarafınıza gönderilecektir.

Başarınızı tebrik eder, saygılar sunarım.

Dr. Hatice Mahur TURAN
Sağlık Bilimleri Araştırma Destek
Grubu (SBAG) Grup Koordinatörü V.

PUAN SEVİYESİ: B

A: Çok İyi B: İyi C: Orta D: İyi Değil E: Yetersiz F: Özgün Değeri Yetersiz

Panel puanı A ve B seviyesinde olan projeler desteklenmiştir.

* Bir kişi kariyer hayatı boyunca 1002-A Hızlı Destek Modülü ve 1002-B Acil Destek Modülü kapsamında toplamda en fazla beş kez proje yürütücüsü olarak görev alabilir.
